# Design and synthesis of a photoswitchable guanidine catalyst

**DOI:** 10.3762/bjoc.8.209

**Published:** 2012-10-24

**Authors:** Philipp Viehmann, Stefan Hecht

**Affiliations:** 1 Department of Chemistry, Humboldt-Universität zu Berlin, Brook-Taylor-Str. 2, 12489 Berlin, Germany

**Keywords:** azobenzenes, guanidines, molecular switches, organocatalysis, photochromism, ring opening polymerization

## Abstract

A novel design as well as a straight-forward synthesis for a photoswitchable guanidine catalyst is reported. Intense studies of the photochromic properties demonstrated the reversible switchability of its photosensitive azobenzene moiety. Its activity in the ring-opening polymerization (ROP) of *rac*-lactide was investigated as well. The obtained results are discussed, and an additional guanidine was synthesized and utilized in the ROP of *rac*-lactide in order to explain the findings.

## Introduction

The macroscopic properties of a given polymer, e.g., the glass-transition temperature, morphology, density and tensile strength, are strongly dependent on its microscopic structure. It is well known that this structure, which is characterized by parameters such as molecular weight and distribution, composition, regiochemistry and stereochemistry (tacticity), critically depends on the interaction between catalyst and monomer during the polymerization process. Gaining control over these processes has therefore been the subject of intense research efforts. With this in mind, chemists have recently started to incorporate gates into catalysts to control their action through external stimuli. The use of light as such an external, noninvasive, and well-controlled stimulus perhaps represents the most attractive approach to controlling catalytic activity and selectivity due to the attainable high spatio-temporal resolution [1]. In order to create such photoswitchable catalysts [2], photochromic moieties [3–5] have to be incorporated into the catalyst system [6–13]. To the best of our knowledge, successful examples of photoswitchable catalysts for the ring-opening polymerization (ROP) of lactide have not been reported thus far, although a remarkable example of a photocaged system has been reported [14].

**Scheme 1:**
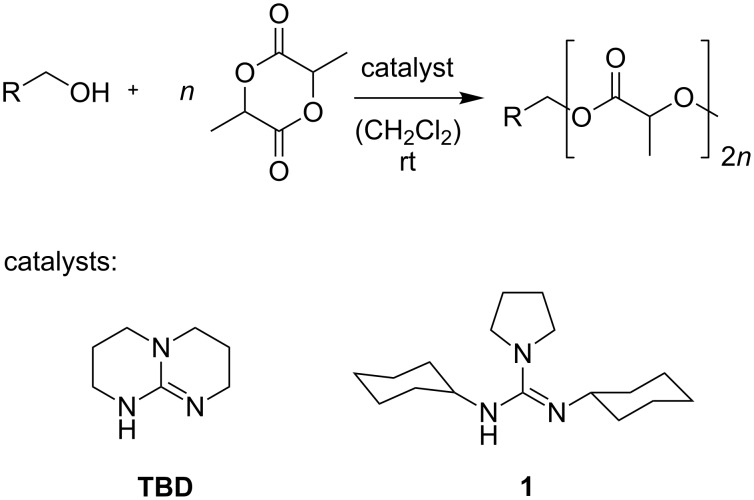
Ring-opening polymerization (ROP) of lactide with TBD or the acyclic guanidine **1** as catalysts [16,18].

Metal-based catalysts as well as organocatalysts have been widely studied in the ROP of lactide [15]. Guanidines, and especially 1,5,7-triazabicyclo[4.4.0]dec-5-ene (TBD), have proven to be very powerful in this context (Scheme 1) [16]. However, due to the discussion of several polymerization mechanisms [17], TBD is a difficult target for the incorporation of photoresponsive switches. Recent work indicates that the activation mechanism for acyclic guanidines, such as guanidine **1** (Scheme 1), is strongly dependent on the formation of hydrogen bridges to monomer and initiator [18]. Note that with pyrenebutanol as the initiator, 200 repeat units are grown within 20 s by using TBD as the catalyst, while 1 h is needed with the acyclic guanidine **1** to reach the same average chain length.

**Scheme 2:**
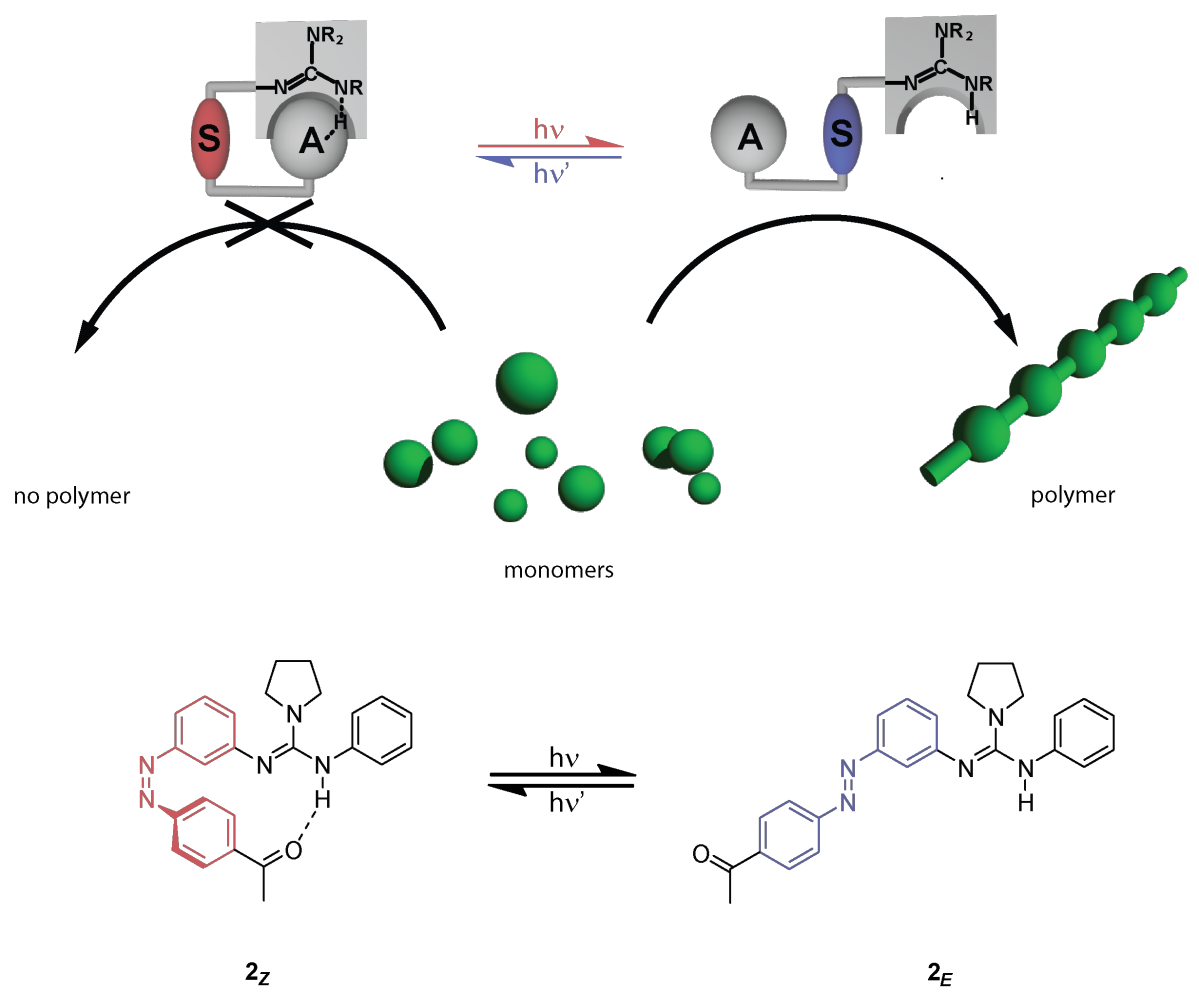
Illustration of a photoswitchable guanidine catalyst for the ROP of lactide and the corresponding target structures **2*_Z_*** and **2*_E_*** (S = switching unit, A = H-bond acceptor).

Based on this finding it should be possible to create a catalyst that may be deactivated by formation of intramolecular hydrogen bonds to an acceptor (A) and activated by light-triggered dissociation of such intramolecular hydrogen bonds (Scheme 2). Herein, we report the synthesis of the first photoswitchable guanidine, as well as its photochromic behavior. Upon irradiation, the incorporated azobenzene is supposed to undergo photoinduced *E*→*Z* isomerization, allowing the Brønsted acceptor, in this case a ketone, to form a hydrogen-bridge to the proton of the guanidine.

## Results and Discussion

**Scheme 3:**
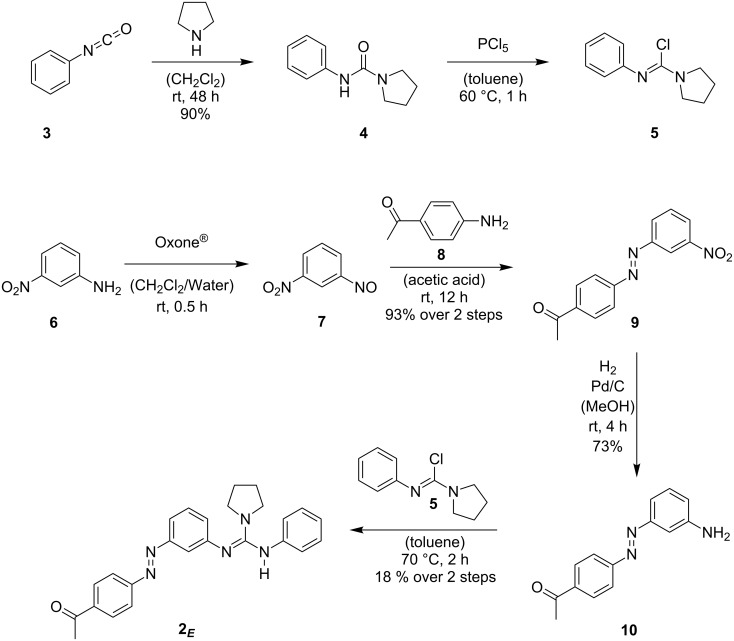
Synthesis of guanidine **2*_E_***.

The synthesis of guanidine **2*_E_*** (Scheme 3) was accomplished starting from phenylisocyanate (**3**) and 3-nitroaniline (**6**). The latter was converted by known procedures into its corresponding nitroso derivative **7** [19], followed by a Mills coupling with 4-aminoacetophenone (**8**) to give azobenzene **9** in 93% yield over two steps. Nitroazobenzene **9** was transformed into its amino derivative **10** by catalytic hydrogenation. Note that under the employed conditions reduction of the azobenzene function of compound **9** was observed, yet the formed hydrazine analogue reoxidized in situ upon being stirred under atmospheric conditions (air). The free amine **10** finally reacts with the in situ prepared Vilsmeyer salt **5** to provide the desired target compound **2*_E_***. The therefore necessary urea **4** can be obtained from the addition of pyrrolidine to phenylisocyanate (**3**).

**Figure 1:**
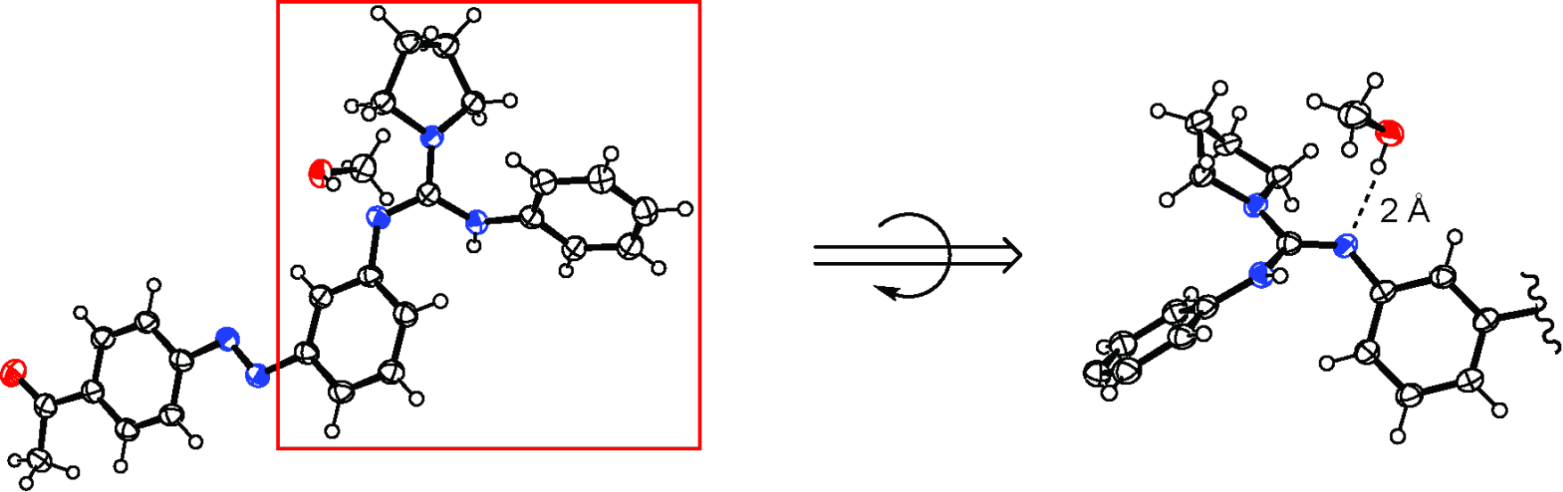
ORTEP image of the single-crystal X-ray structure of guanidine **2*_E_***, as well as a rotated close-up of the guanidine moiety, showing 50% thermal ellipsoids (CCDC 891016).

The structure of guanidine **2*_E_*** was proven by NMR spectroscopy, HRMS, as well as by single-crystal X-ray structure analysis (Figure 1). To our knowledge, this is the first example of an X-ray analysis of an acyclic guanidine–methanol adduct, clearly proving the existence of a hydrogen bridge between an alcohol OH function and the nitrogen of the guanidine. This significantly supports the mechanism of alcohol activation in the polymerization process. Additionally, the crystal structure confirms the validity of our concept, illustrating the azobenzene’s utilization as a spacer by spatial separation of the guanidine core and carbonyl function.

**Figure 2:**
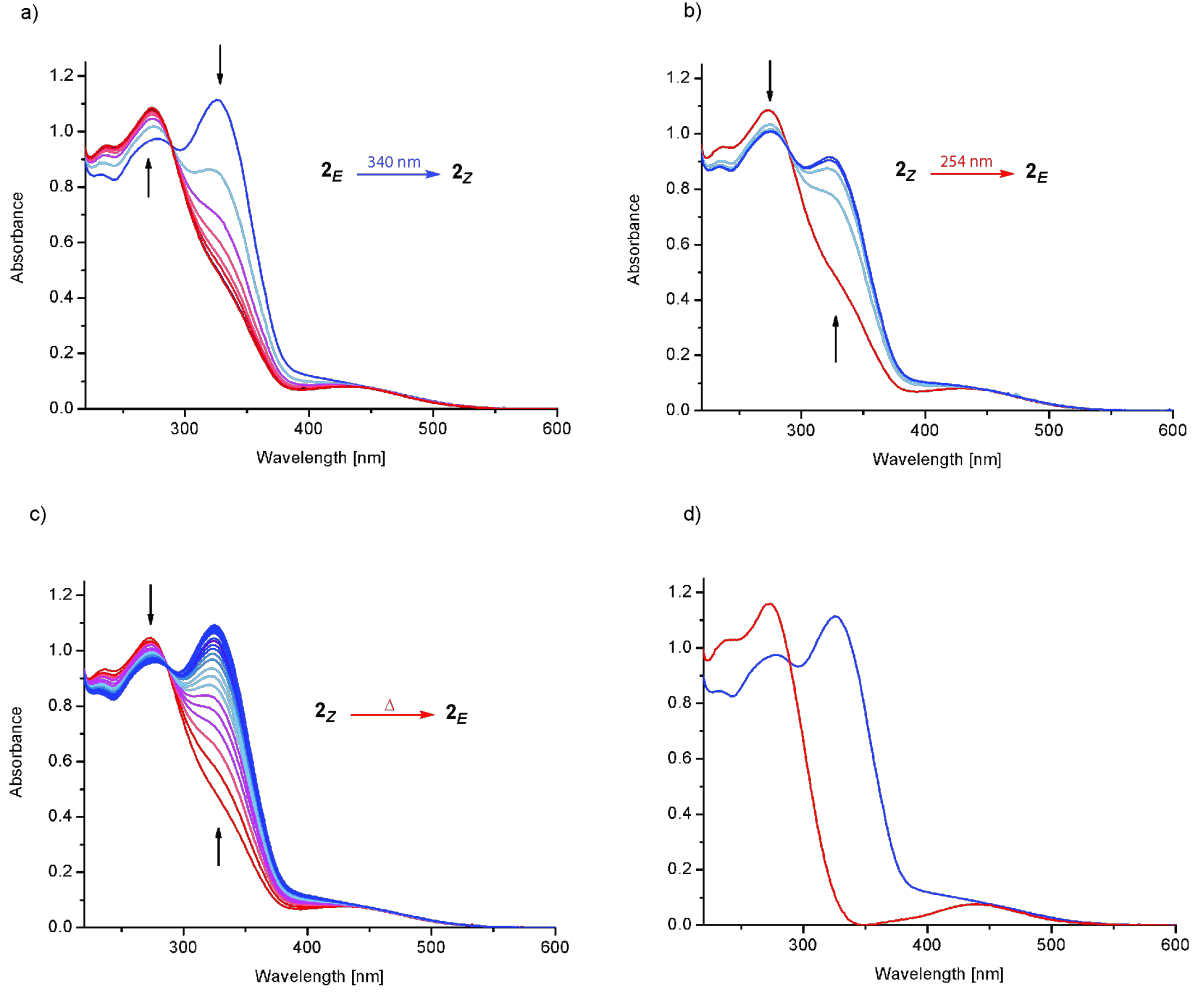
UV–vis spectra of guanidine **2** in acetonitrile, *c* = 3.9·10^−5^ mol/L. (a) *E*→*Z* isomerization with irradiation at 340 nm, 25 °C; (b) *Z*→*E* isomerization with irradiation at 254 nm, 25 °C; (c) thermal *Z*→*E* isomerization at 40 °C; (d) spectra of **2*_E_*** (blue) and **2*_Z_*** (calculated, red).

In order to utilize the incorporated light-sensitive azobenzene functionality, the photochromic behavior of guanidine **2** was investigated by UV–vis spectroscopy (Figure 2). The UV–vis spectrum of guanidine **2*_E_*** in acetonitrile exhibits the expected behavior, similar to the unsubstituted parent azobenzene, i.e., a low intensity n→π* band in the visible region and a much stronger π→π* band in the UV region of the spectrum. Note that the n→π* band partially overlaps with the π→π* band in the (*E*)-isomer. Upon irradiation at 340 nm (Figure 2a), the π→π* band of the isomer **2*_E_*** at 326 nm vanishes and the π→π* band of the isomer **2*_Z_*** with its blue-shifted maximum at 273 nm appears. The content of isomer **2*_Z_*** in the photostationary state (pss) was determined to amount to 61% by UPLC using a detection wavelength on the isosbestic point (290 nm). Based on this data, the UV–vis spectrum of the isomer **2*_Z_*** was calculated (Figure 2d), assuming that the total absorption is the sum of the partial absorptions. Obviously, the significant hypsochromic shift of the π→π* band leads to a separation of the n→π* and π→π* bands for the (*Z*)*-*isomer. As expected, an inverse behavior is observed upon irradiation at 254 nm, inducing photoisomerization of guanidine **2*_Z_*** back to its precursor **2*_E_*** (Figure 2b). The amount of isomer **2*_E_*** in the resulting pss was determined to be 94%. The same nearly quantitative *Z*→*E* photoisomerization, giving 92% of **2*_E_*** in the pss, is obtained upon irradiation at 430 nm.

Related to the supposed function of guanidine as a switchable catalyst, these experiments demonstrate the reversible photoisomerization of the azobenzene moiety. Nevertheless, the switching properties can still be improved, in particular to obtain a pss with higher (*Z*)-content in the *E*→*Z* photoisomerization. Besides the photo-induced *Z*→*E* isomerization, the thermal back-reaction of guanidine **2*_Z_*** was examined as well (Figure 2c). Based on the measured UV–vis and UPLC data and assuming a first-order rate law, the rate constant of the thermal *Z*→*E* isomerization at 40 °C was determined to be *k*_(40 °C)_ = 6.3·10^−5^ s^−1^ corresponding to a half-life of τ_1/2_ = 3 h. The measurement at slightly elevated temperature (40 °C) was necessary since at 25 °C the thermal half-life increases significantly (Supporting Information File 1, Figure S-5), leading to distinct difficulties in its determination, e.g., the evaporation of the solvent and the resulting change in concentration. Therefore, the rate constant and thermal half-life at 25 °C could only be estimated by using data points from the first 36 h (Supporting Information File 1, Figure S-6) yielding *k*_(25 °C)_ = 4.2·10^−6^ s^−1^ and τ_1/2_ = 46 h. Consequently, the half-life significantly exceeds the expected duration of the polymerization reaction [18], rendering guanidine **2** very attractive for this purpose. In summary, the observed photochromic behavior detailed above indicates promising properties for the utilization of guanidine **2** as a photoswitchable catalyst.

**Scheme 4:**
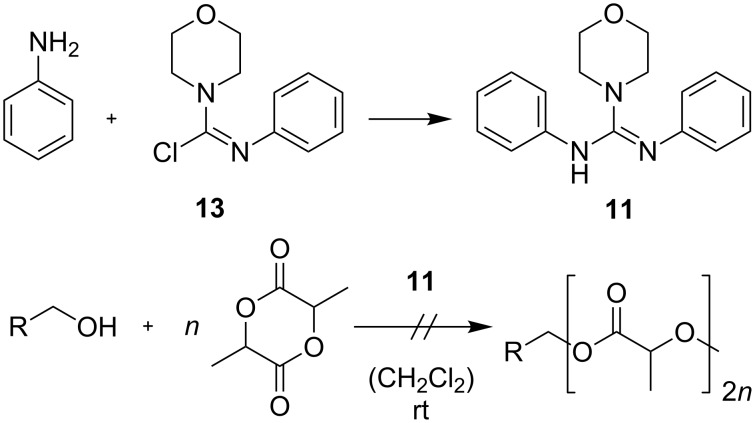
Guanidine **11** as a catalyst in the ROP of *rac*-lactide (catalyst/initiator/monomer ratio = 10:1:100).

In order to determine the activity of the catalyst, the crystal-bound methanol was removed and guanidine **2*_E_*** was utilized in the ROP of *rac*-lactide with pyrenebutanol as the initiator. In a catalyst/initiator/monomer ratio of 1:1:100 no significant formation of polylactide was observed at rt even after 48 h. The absence of reactivity for guanidine **2*_E_*** in the ROP of *rac*-lactide may have various origins. First of all, it is obvious that in comparison with catalytically active guanidine **1**, the two cyclohexane substituents on the nitrogen atoms of guanidine are replaced by aromatic residues, which, at least in one case, is necessary for connecting the photochromic azobenzene moiety to the guanidine core. This replacement most likely results in a significant reduction of the basicity of guanidine. Additionally, the acceptor ketone moiety itself may interact with the initiator or the active end of the polymer chain, thereby interfering with catalysis. In order to examine the latter, guanidine **11** was synthesized from Vilsmeyer salt **13** and aniline and tested in the ROP of *rac*-lactide (Scheme 4). The morpholino substituent was introduced to improve the crystallization behavior of guanidine and should not affect the polymerization.

**Figure 3:**
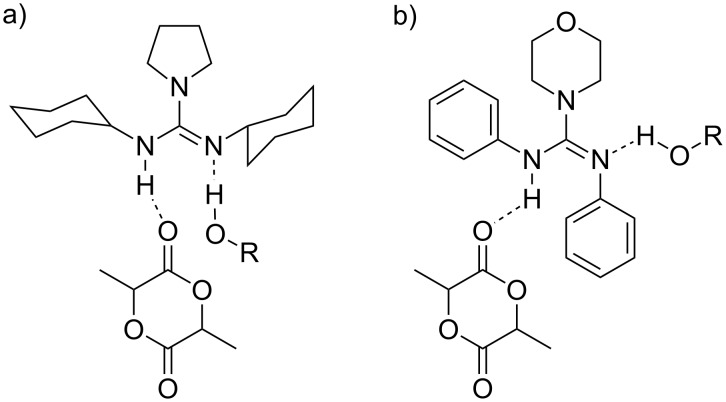
Supposed intermediates resulting from either a cyclohexane-substituted guanidine (a) [18] or an aromatic substituted guanidine (b) with an alcohol and lactide.

In a catalyst/initiator/monomer ratio of 10:1:100 with pyrenebutanol as initiator no significant formation of polylactide was observed at rt even after 48 h. This indicates that the catalytic inactivity of guanidine **2*_E_*** is indeed caused by the guanidine core itself. Besides the previously mentioned basicity issue, a possible reason for this behavior may be discerned from the single-crystal X-ray structure analysis of guanidine **2*_E_***. It can be seen that, in contrast to the supposed polymerization mechanism (Figure 3a) [18], the lone pair of the nitrogen and the hydrogen of the adjacent nitrogen are not arranged in a parallel manner, i.e., unidirectional facing the substrates (Figure 3b). If the observed conformation is not caused by stacking effects in the solid state and prevails in solution, it may substantially lower the catalytic activity of the guanidines **2*_E_*** and **11**.

## Conclusion

In summary, we developed a new approach to the design and synthesis of the novel, photoswitchable guanidine **2**. After detailed investigation of its photochromic properties and demonstrating its reversible, light-induced switchability, we started to investigate the reasons for its inactivity in the ROP of lactide. Our efforts are now directed toward the improvement of the design of the catalyst by exchanging one aromatic residue with an alkyl substituent to improve the basicity of guanidine, as well as by introducing sterically crowded substituents or ring constraints in order to direct the position of the relevant lone pair of guanidine as well as the NH-function.

## Supporting Information

The crystal structure has been deposited at the Cambridge Crystallographic Data Centre and allocated the deposition number CCDC 891016.

File 1 Experimental details and spectra.
