# Supplementary material for: Design and synthesis of a photoswitchable guanidine catalyst
Source: Beilstein J Org Chem. 2012 Oct 24;8:1825–30. doi: 10.3762/bjoc.8.209 (PMC3511018; doi:10.3762/bjoc.8.209)
Supplement: File 1 — Experimental details and spectra. [file Beilstein_J_Org_Chem-08-1825-s001.pdf]

Supporting Information

for

Design and synthesis of a photoswitchable guanidine catalyst

Philipp Viehmann and Stefan Hecht*

Address: Department of Chemistry, Humboldt-Universität zu Berlin, Brook-Taylor-Str. 2, 12489 Berlin, Germany

Email: Stefan Hecht - sh@chemie.hu-berlin.de

* Corresponding author

Experimental details and spectra

Table of contents:

General experimental details	S2
Synthesis	S3
UV–vis absorption spectra	S7
NMR-Spectra	S10
Single-crystal X-ray structure analysis of guanidine $\mathbf{2}_{E}$	S14
Reference	S15

General experimental details

Solvents and commercial starting materials were used as supplied. The solvents were dried before use, if necessary, employing an Innovative Technologies solvent-purification system (multi-unit micro series). Silica gel for chromatography from Merck (0.035–0.070 mm, 60 Å) was used for column chromatography. The petroleum ether (PE) used had a boiling range of 40–60 °C. NMR spectra were recorded on a 500 MHz (125 MHz for ^{13}C) Bruker AVANCE II 500 spectrometer or on a 300 MHz (75.6 MHz for ^{13}C) Bruker DPX 300 spectrometer at 25 °C using residual protonated solvent signals as internal standards (^{1}H: δ($CHCl_3$) = 7.26 ppm, ^{13}C: δ($CHCl_3$) = 77.16 ppm). Ultrahigh-performance liquid chromatography/mass spectrometry (UPLC/MS) was performed on a Waters Acquity UPLC equipped with a Waters LCT Premier XE Mass detector for high-resolution MS (HRMS, ESI^+-ionization) and with Waters Alliance systems (consisting of a Waters Separations Module 2695, a Waters Diode Array Detector 996 and a Waters Mass Detector ZQ 2000). TLC was performed on Merck Silica Gel 60 F_{254} TLC plates with a fluorescent indicator employing a 254 nm UV-lamp for visualization. All procedures linked to photochemistry were performed with spectrophotometric grade solvents. UV–vis spectroscopy was performed on either a Varian Cary 50 or Varian Cary 60 UV–vis spectrophotometer equipped with a Peltier thermostated cell holder at 25 ± 0.05 °C. Analytical irradiation experiments were performed on degassed solutions (Ar for 5 min, 2–4·10^{-5} M) in CH_3CN by using a LOT-Oriel 1000 W high-pressure xenon lamp (XBO) equipped with two cut-off filters resulting in a narrow spectral window ($\lambda_{max\,T}$ = 340 nm @ 35% T, FWHM = 42 nm) or interference filter with maxima at 254 nm respectively 430 nm.

Synthesis

N-phenylpyrrolidine-1-carboxamide (4)

A solution of phenylisocyanate (5.956 g, 50.00 mmol, 1 equiv) was cooled to 0 °C. Pyrrolidine (3.556 g, 50.00 mmol, 1 equiv) was added carefully over 20 min. After the addition was completed, the mixture was warmed to rt and stirred for 48 h. After the reaction was completed the solvent was removed in vacuo. The resulting solid was washed with PE and dried in vacuo. The product was isolated in 95% yield (9.002 g, 47.50 mmol) as a white solid. ^{1}H NMR (500 MHz, $CDCl_3$): δ = 7.41 (d, $2H_{aryl}$, ^{3}J = 8.5 Hz), 7.26 (dd, $2H_{aryl}$, ^{3}J = 7.8 Hz, , ^{3}J = 8.5 Hz), 7.00 (dd, $1H_{aryl}$, ^{3}J = 7.8 Hz), 6.25 (s, N-H), 3.44 (t, $4H_{CH2}$, ^{3}J = 6.5 Hz), 1.94 (t, $4H_{CH2}$, ^{3}J = 6.5 Hz) ppm. ^{13}C NMR (125 MHz, $CDCl_3$): δ = 154.2, 139.4, 129.0, 122.9, 119.7, 46.0, 25.8 ppm. ESI-HRMS: calcd for $C_{11}H_{15}N_2O^+$, 191.1184; found, 191.1106.

(*Z*)-*N*-phenylpyrrolidine-1-carbimidoyl chloride (5)

A mixture of urea **4** (0.190 g, 1.00 mmol, 1 equiv) and PCl_5 (0.312 g, 1.50 mmol, 1.5 equiv) in 25 mL dry toluene was heated to 60 °C for 1 h until a white solid precipitated and TLC indicated total conversion of the urea. After that, the solvent was removed in vacuo. The resulting white solid was used immediately without any further purification.

(*E*)-3-Nitro-4’-acetoazobenzene (9)

1-Nitro-3-nitrosobenzene (**7**) [1] (5.400 g, 35.50 mmol, 2 equiv) was suspended in glacial AcOH (350 mL) and the suspension was sonicated until a clear green solution was obtained. *p*-Acetoaniline (**8**, 2.399 g, 17.75 mmol, 1 equiv) was added, and the

mixture was stirred at rt for 12 h. 500 mL of water was added and the resulting precipitate was separated by filtration using a suction filter and washed with water (300 mL). The solid was recrystallized from methanol to afford the product in 93% yield (8.903 g, 16.51 mmol) as an orange solid. ^{1}H NMR (300 MHz, $CDCl_3$): δ = 8.76 (s, H_{aryl}), 8.30 (d, ^3J = 8.1 Hz, H_{aryl}), 8.37 (d, ^3J = 8. Hz, H_{aryl}), 8.03 (d, 2H_{aryl}, ^3J = 8.7 Hz), 8.14 (d, 2H_{aryl}, ^3J = 8.7 Hz), 7.74 (dd, H_{aryl},^3J = 8.1 Hz, ^3J = 8.1 Hz), 2.68 (s, 3H_{CH3}) ppm. ^{13}C NMR (75 MHz, $CDCl_3$): δ = 197.5, 154.4, 153.0, 139.5, 130.1, 129.5, 129.5, 125.6, 123.4, 117.2, 26.9 ppm. ESI-HRMS: calcd for $C_{14}H_{12}N_3O_3^+$, 270.0879; found, 270.0883.

(*E*)-3-Amino-4’-acetoazobenzene (10)

The nitro compound **9** (0.539 g, 2.00 mmol, 1 equiv) was suspended in 50 mL of MeOH and stirred for about 15 min. Then 50 mg Pd/C where carefully added. The setup was completed by the attachment of a H_2 balloon. The mixture was stirred for 4 h under the H_2 atmosphere until TLC indicated complete consumption of the starting material, then the green mixture was stirred overnight under air atmosphere to reoxidize the azobenzene functionality. After completion of the stirring, the crude mixture was filtered through silica with methanol as eluent. The solvent was removed in vacuo and the crude product recrystallized from methanol. The product was obtained in 73% yield (0.350 g, 1.46 mmol) as red crystals. ^{1}H NMR (300 MHz, $CDCl_3$): δ = 8.14 (d, 2H_{aryl}, ^3J = 8.6 Hz), 7.92 (d, 2H_{aryl}, ^3J = 8.6 Hz), 7.26 (dd, H_{aryl}, ^3J = 7.9 Hz), 7.08–7.19 (m, H_{aryl}, H_{aryl}), 6.81 (d, H_{aryl}, ^3J = 7.9 Hz), 5.50 (s, NH_2), 2.64 (s, 3H_{CH3}) ppm. ^{13}C NMR (75 MHz, $CDCl_3$): δ = 197.4, 154.4, 153.1, 149.8, 138.1, 129.8, 129.6, 122.5, 118.1, 113.1, 105.3, 27.0 ppm. ESI-HRMS: calcd for $C_{14}H_{14}N_3O$, 240.1137; found, 240.1153.

Guanidine 2

The Vilsmeyer salt **5** (0.209 g, 1.00 mmol, 1 equiv) was dissolved in 10 mL dry toluene and triethylamine (0.4 mL, 3 mmol, 3 equiv) was added carefully. After the addition was completed, azo amino compound **10** (0.235 g, 1.00 mmol, 1 equiv) was added in one portion and the mixture was heated to 70 °C. After UPLC showed complete consumption of the amine **10** the mixture was cooled to rt and the resulting red solid was filtered. The solid was then washed with toluene whereas the filtrate was collected separately. The toluene was removed in vacuo and the resulting solid was purified three times by column chromatography (PE/EE 7:3). After removal of the solvent the red solid was recrystallized four times from methanol, yielding 18% (0.076 g, 0.18 mmol) of the desired product. ^{1}H NMR (500 MHz, CD_2Cl_2): δ = 8.27 (d, 2H_{aryl}, ^3J = 8.4 Hz), 8.1 (d, 2H_{aryl}, ^3J = 8.4 Hz), 7.69 (d, H_{aryl}, ^3J = 7.5 Hz), 7.61 (s, H_{aryl}), 7.54 (dd, H_{aryl}, ^3J = 8.0 Hz, ^{3}J = 6.8 Hz), 7.40 (dd, 2H_{aryl}, ^3J = 7.5 Hz, ^3J = 8.0 Hz), 7.20 (d, H_{aryl}, ^3J = 7.5 Hz), 7.01–7.16 (m, H_{aryl}, H_{aryl}), 5.93 (s (br), NH), 3.59 (t, 4H_{CH2}, ^3J = 6.8 Hz), 2.83 (s, 3H_{CH3}), 2.07 (t, 4H_{CH2}, ^3J = 6.8 Hz) ppm. ^{13}C NMR (125 MHz, CD_2Cl_2): δ = 197.3, 155.1, 153.7, 138.5, 129.8, 129.4, 129.2 122.8, 122.0, 47.8, 26.8, 24.4 ppm. ESI-HRMS: calcd for $C_{25}H_{26}N_5O$, 412.2137; found, 412.2106.

***N*-Phenylmorpholine-4-carboxamide (12)**

A solution of phenylisocyanate (5.956 g, 50.00 mmol, 1 equiv) was cooled to 0 °C, morpholine (4.356 g, 50.00 mmol, 1 equiv) was added carefully over 20 min. After the addition was completed, the mixture was warmed to rt and stirred for 48 h. After the reaction was completed the solvent was removed in vacuo. The resulting solid was washed with PE and dried in vacuo. The product was isolated in quantitative yield (10.298 g, 50.00 mmol) as a white solid. ^{1}H NMR (500 MHz, $CDCl_3$): δ = 7.33 (d,

2H_{aryl}, ^3J = 8.5 Hz), 7.26 (dd, 2H_{aryl}, ^3J = 7.3 Hz, ^3J = 8.5 Hz), 7.03 (t, H_{aryl}, ^3J = 7.3 Hz), 6.69 (s, N-H), 3.66 (t, 4H_{CH2}, ^3J = 5.0 Hz), 3.42 (t, 4H_{CH2}, ^3J = 5.0 Hz) ppm.^{13}C NMR (125 MHz, $CDCl_3$): δ = 155.5, 139.0, 129.1, 123.6, 120.6, 66.7, 44.5 ppm. ESI-HRMS: calcd for $C_{11}H_{15}N_2O_2$, 207.1134; found, 207.1129.

_N_-Phenylpyrrolidine-1-carbimidoyl chloride (13)

A mixture of the urea **12** (1.031 g, 5.00 mmol, 1 equiv) and PCl_5 (1.041 g, 5 mmol, 1 equiv) in dry toluene was heated to 60 °C for 1 h until a white solid precipitated and TLC indicated total conversion of the urea. After that, the solvent was removed in vacuo. The resulting white solid was used immediately without any further purification.

_N_,_N'_-Diphenyl-morpholinoguanidine (11)

The Vilsmeyer salt **13** (1.123 g, 5.00 mmol, 1 equiv) was dissolved in 100 mL dry toluene. Aniline (0.235 g, 1.00 mmol, 1 equiv) was added in one portion and the mixture was heated to 70 °C. After the UPLC showed complete consumption of the aniline the mixture was cooled to rt. The solvent was removed in vacuo and the resulting solid was recrystallized from MeOH to give guanidine **11** in 80% yield (1.121 g, 4.00 mmol). ^{1}H NMR (500 MHz, $CDCl_3$): δ = 7.18–7.12 (m, 4-H_{aryl}), 6.92–6.80 (m, 6H_{aryl}), 5.26 (s, br, NH), 3.58 (t, 4H_{CH2}, ^3J = 5.0 Hz), 3.24 (t, 4H_{CH2}, ^3J = 5.0 Hz) ppm. ^{13}C NMR (125 MHz, $CDCl_3$): δ = 151.3, 129.7, 128.7, 123.0, 66.7, 47.41 ppm. ESI-HRMS: calcd for $C_{17}H_{20}N_3O^+$, 282.1606; found, 282.1646.

UV–vis Absorption Spectra

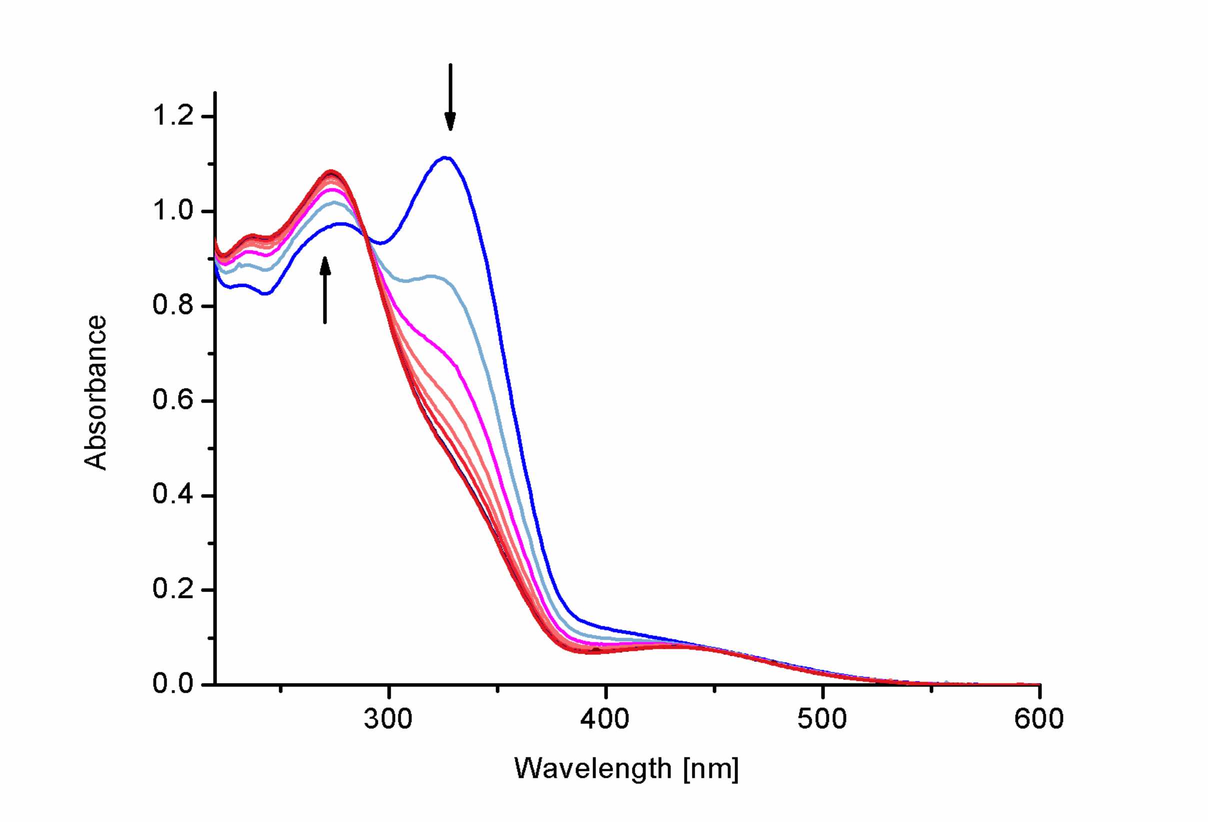

Figure S-1: UV–vis spectra of guanidine **2**, *c* = $3.9 \cdot 10^{-5}$ mol/L, 25 °C, *E*→*Z* isomerization with irradiation at 340 nm.

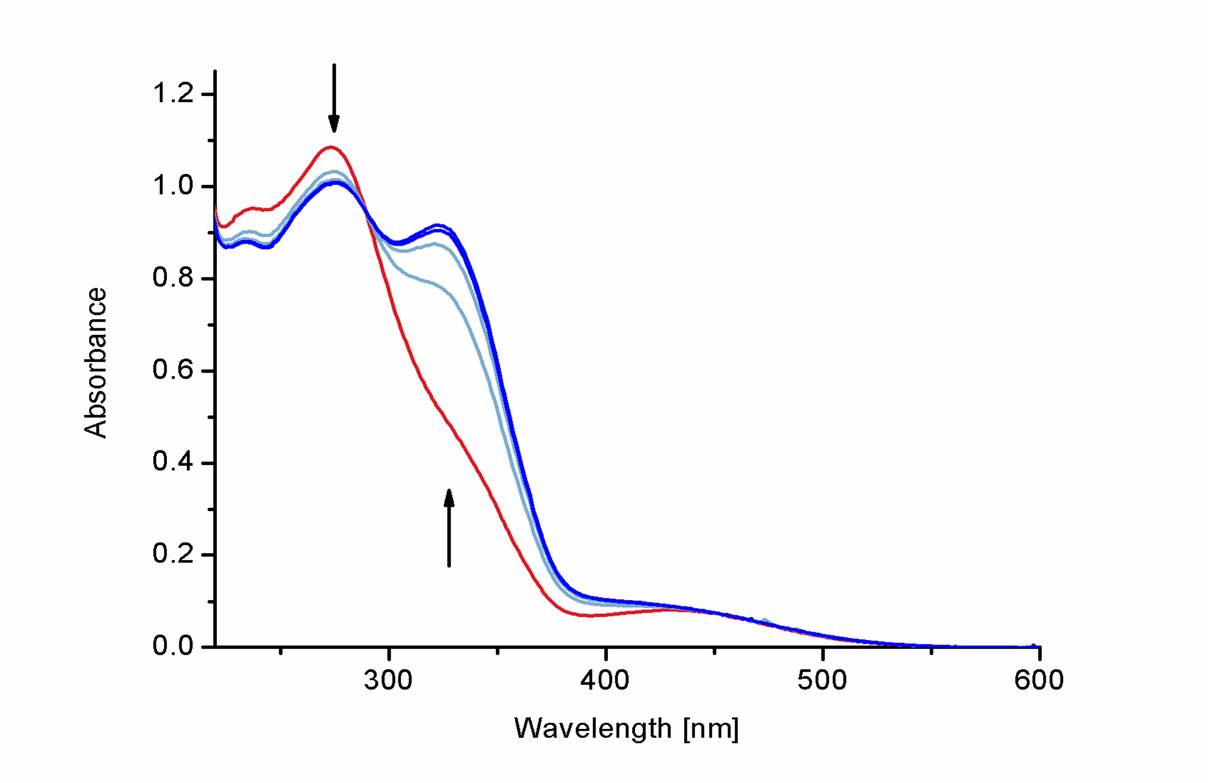

Figure S-2: UV–vis spectra of guanidine **2**, *c* = $3.9 \cdot 10^{-5}$ mol/L, 25 °C, *Z*→*E* isomerization with irradiation at 254 nm.

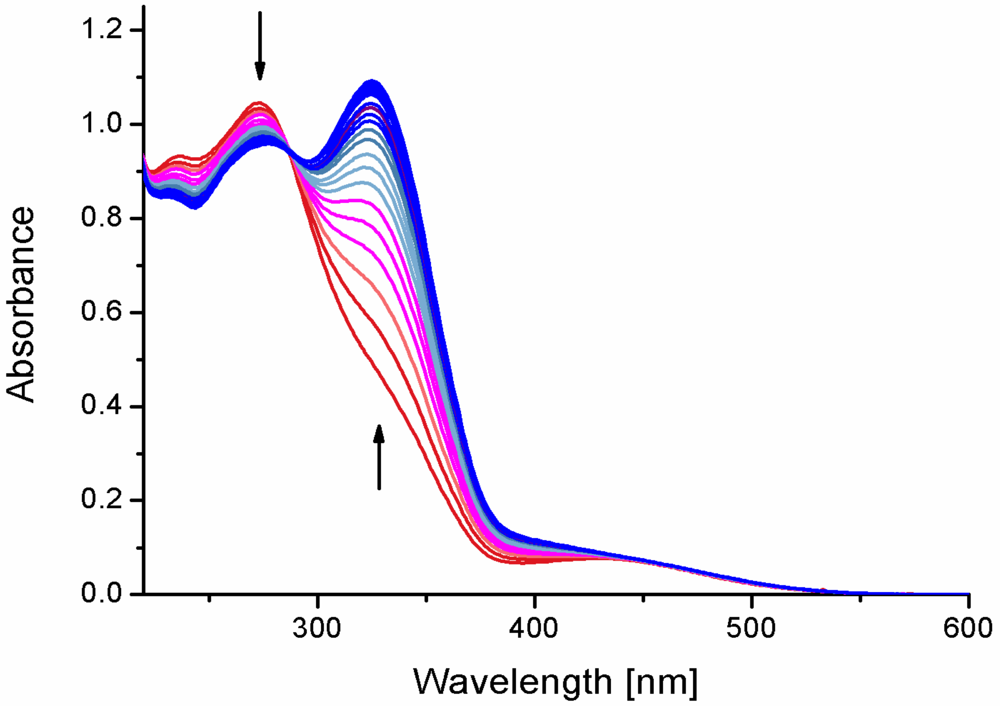

Figure S-3: UV–vis spectra of guanidine **2**, c = 3.9·10^{-5} mol/L, 40 °C, thermal Z→E isomerization.

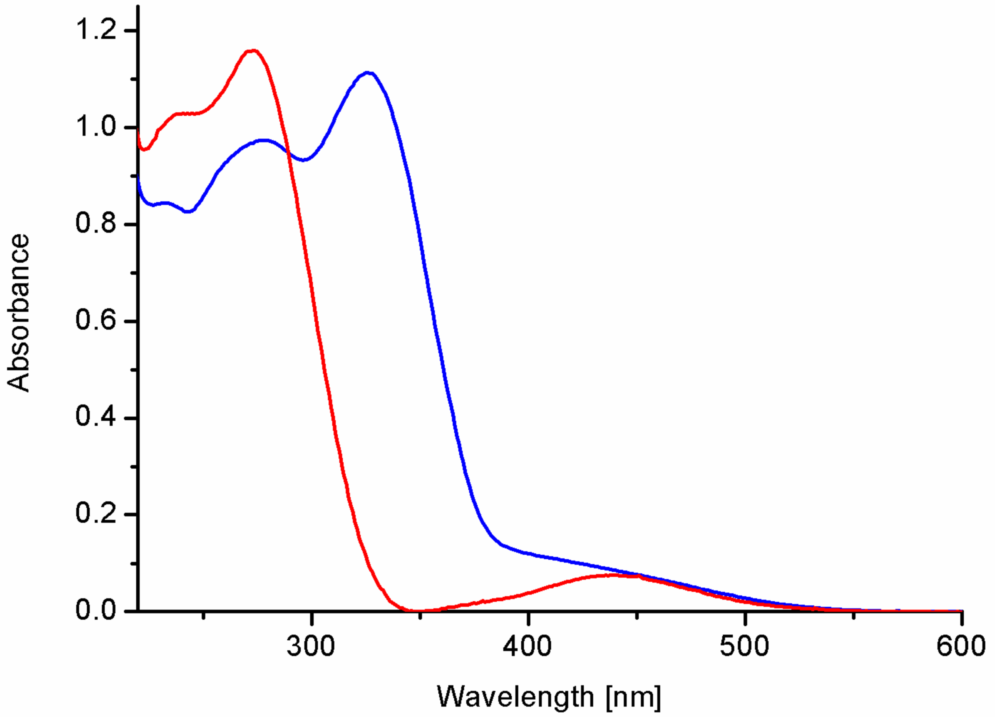

Figure S-4: UV–vis spectra of guanidine **2**$_E$ (blue) and **2**$_Z$ (calculated, red).

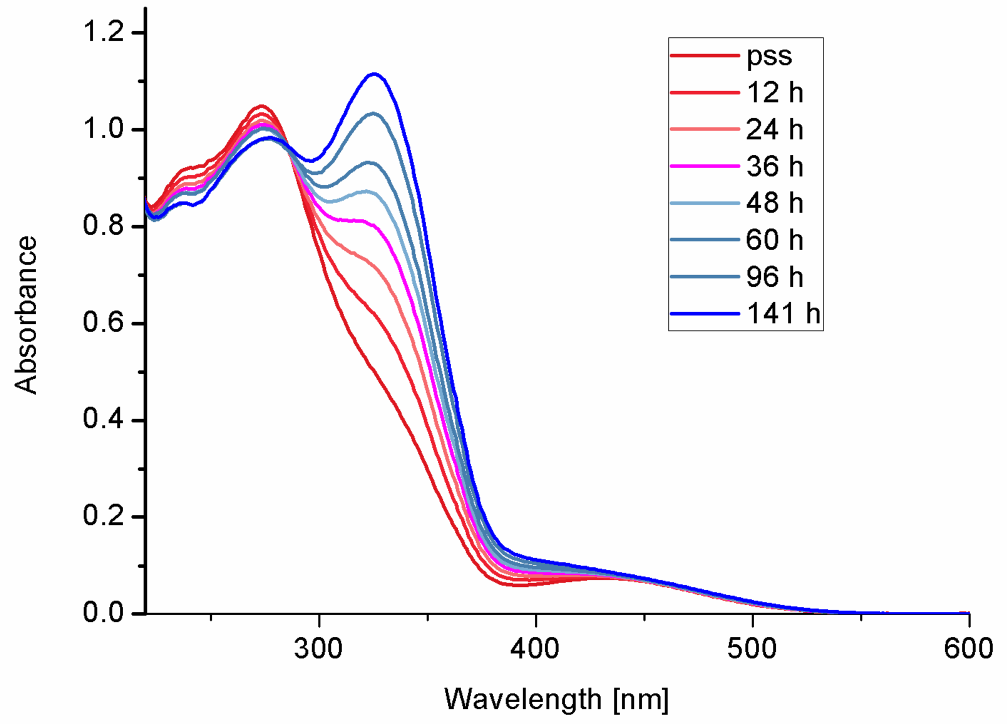

Figure S-5: UV–vis spectra of guanidine 2, $c = 3.9 \cdot 10^{-5}$ mol/L, 25 °C, thermal $Z \rightarrow E$ isomerization.

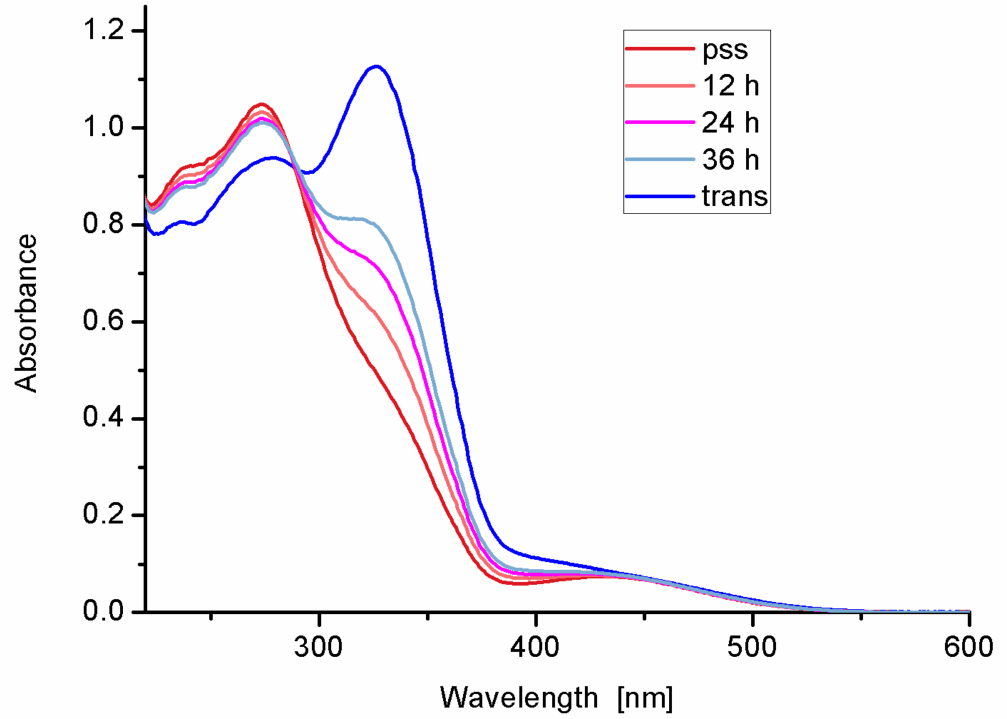

Figure S-6: UV–vis spectra of guanidine 2, $c = 3.9 \cdot 10^{-5}$ mol/L, 25 °C, first 36 h of the thermal $Z \rightarrow E$ isomerization.

NMR-Spectra

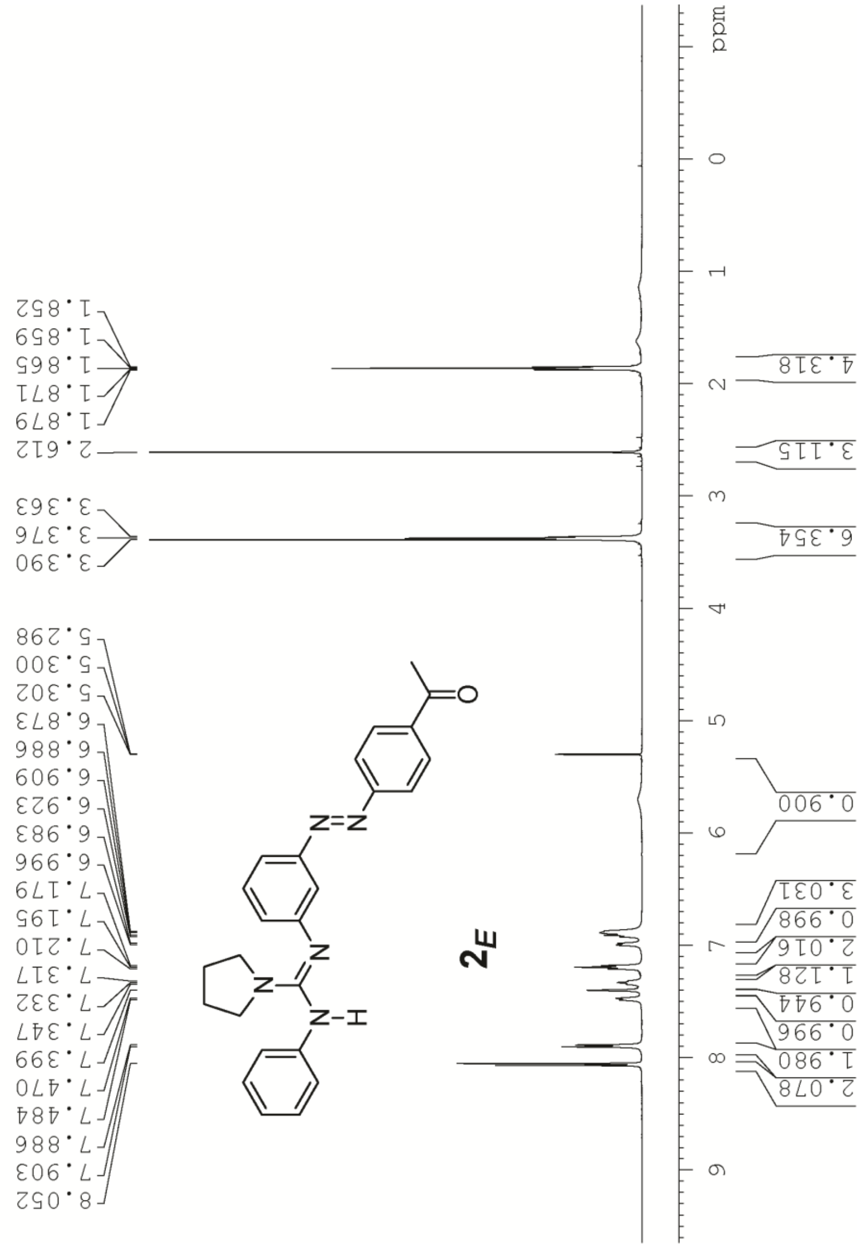

Figure S- 7: ^{1}H NMR spectrum of guanidine **2_E** (CD_2Cl_2, rt).

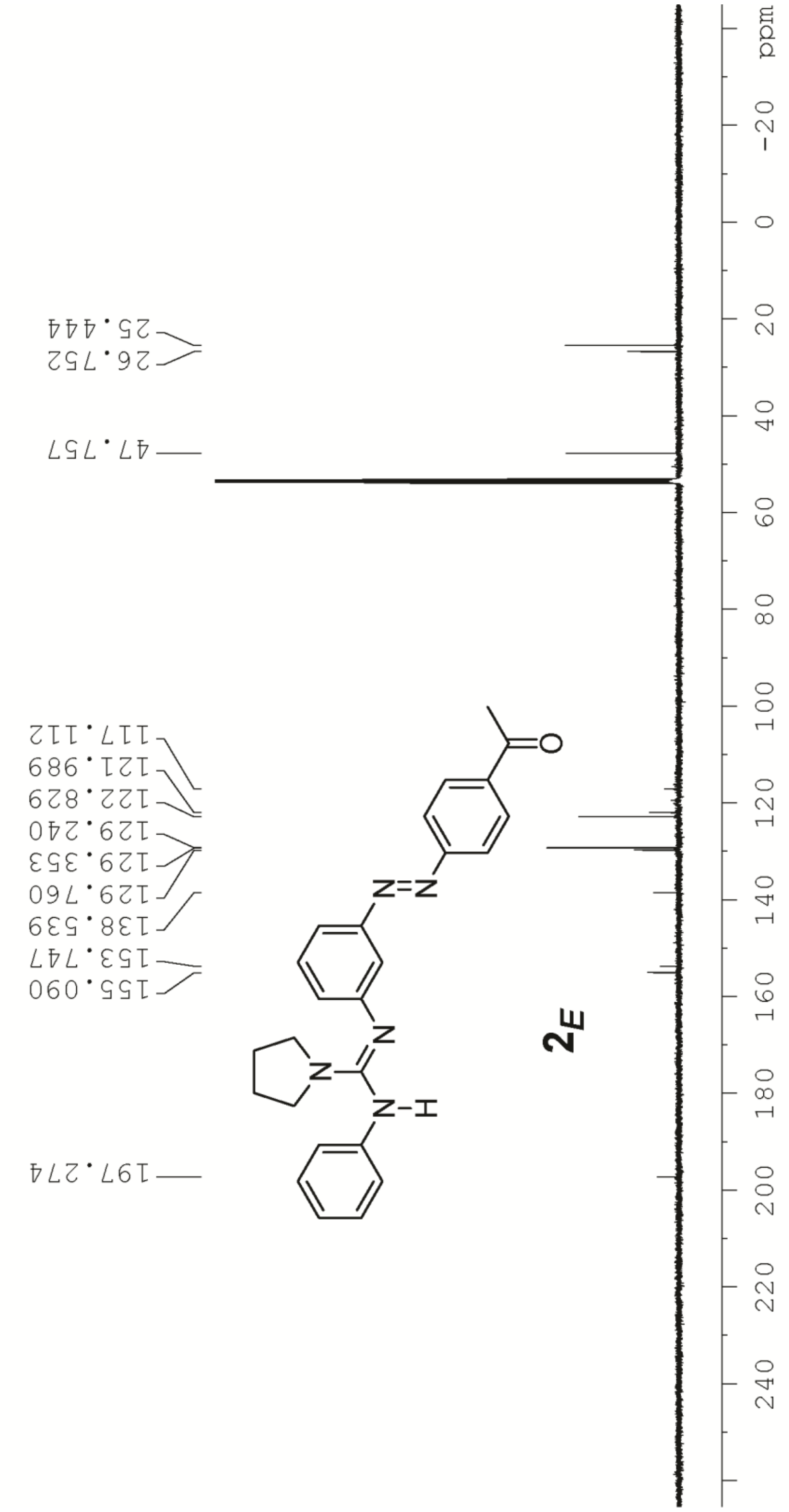

Figure S- 8: ^{13}C NMR spectrum of guanidine $\mathbf{2}_{\boldsymbol{E}}$ (CD_2Cl_2, rt).

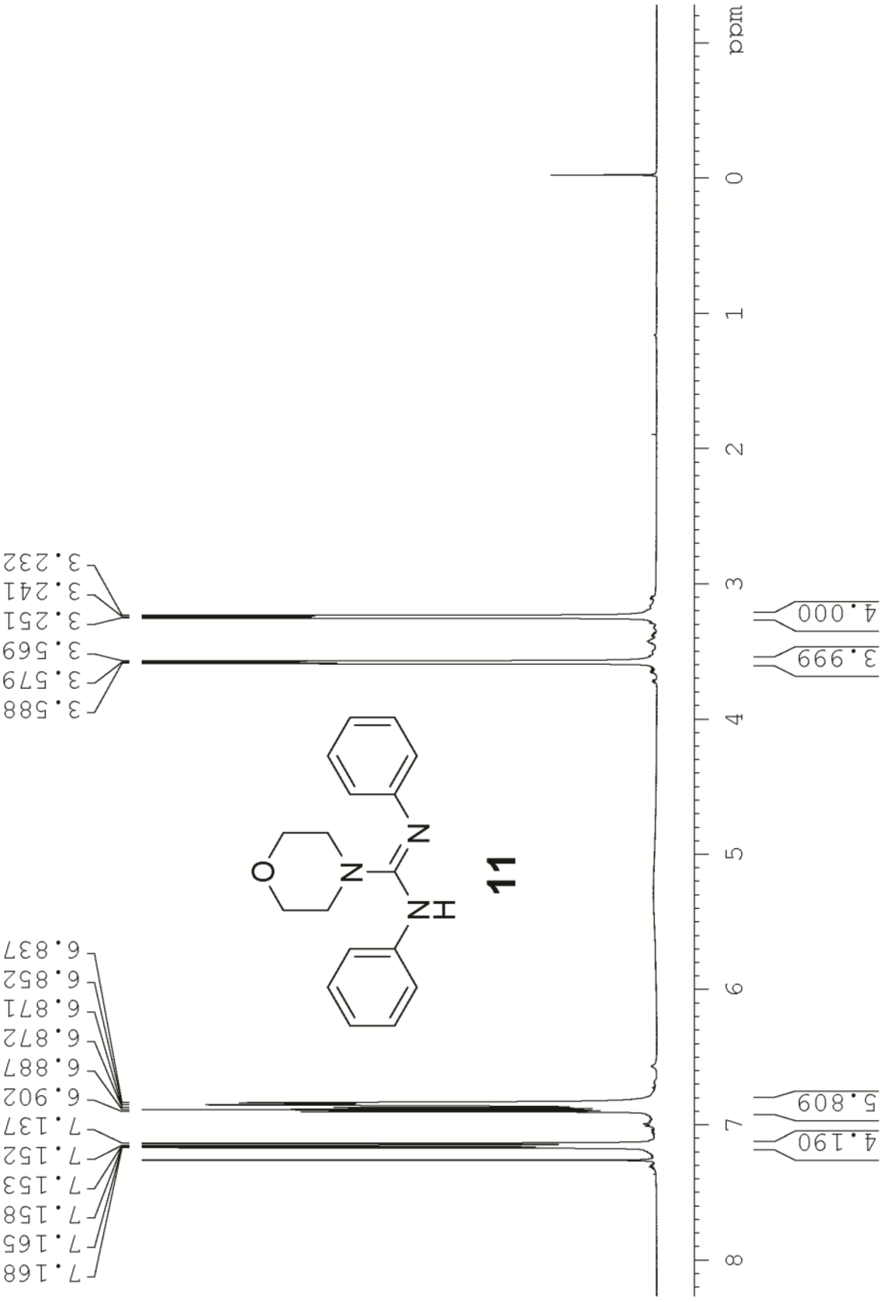

Figure S- 9: ^{1}H NMR spectrum of guanidine **11** ($CDCl_3$, rt).

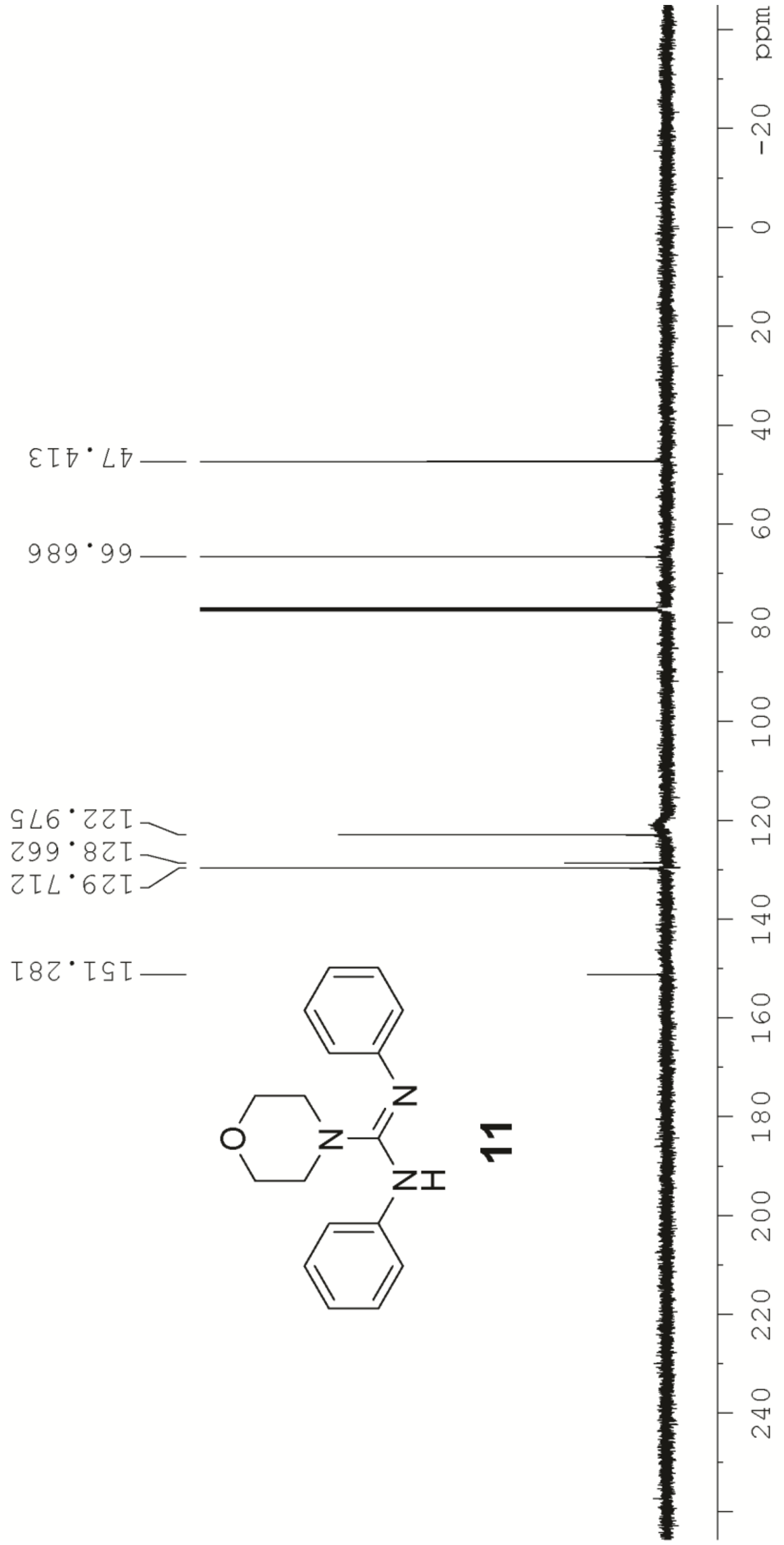

Figure S- 10: ^{13}C NMR spectrum of guanidine $\mathbf{2}_{\boldsymbol{E}}$ ($CDCl_3$, rt).

Single-crystal X-ray structure analyses of guanidine $\mathbf{2_E}$

CCDC 891016 contains the detailed crystallographic data. These data can be obtained free of charge via www.ccdc.cam.ac.uk/data_request/cif, or by emailing data_request@ccdc.cam.ac.uk, or by contacting The Cambridge Crystallographic Data Centre, 12, Union Road, Cambridge CB2 1EZ, UK; fax: +44 1223 336033.

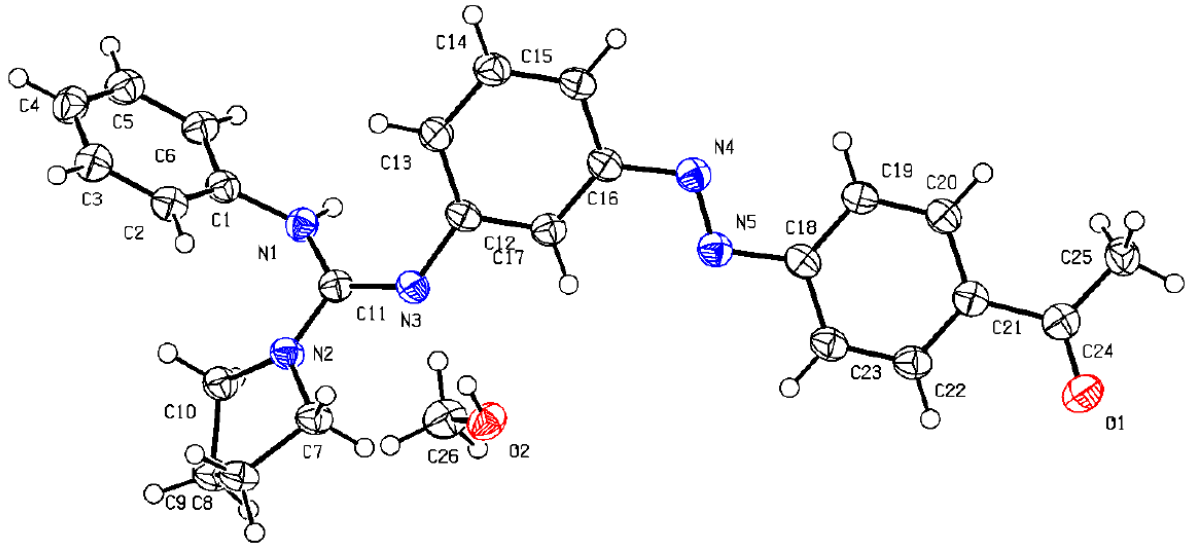

Figure S- 11: ORTEP image of single-crystal X-ray structure analyses of guanidine $\mathbf{2_E}$.

Table 1: Crystal data and structure refinement for guanidine $\mathbf{2_E}$.

Identification code	c:va114
Empirical formula	C26 H29 N5 O2
Formula weight	443.54
Temperature	100(2) K
Wavelength	0.71073 A
Crystal system, space group	Orthorhombic, P c a 21
Unit cell dimensions	a = 16.9368(13) A alpha = 90 deg.
	b = 13.8269(13) A beta = 90 deg.
	c = 9.7260(7) A gamma = 90 deg.

Volume	2277.7(3) A^3
Z, Calculated density	4, 1.293 Mg/m^3
Absorption coefficient	0.084 mm^-1
F(000)	944
Crystal size	0.50 x 0.35 x 0.07 mm
Theta range for data collection	3.51 to 27.50 deg.
Limiting indices	-21<=h<=21, -17<=k<=17, -12<=l<=12
Reflections collected / unique	22278 / 2755 [R(int) = 0.0987]
Completeness to theta = 27.50	99.6 %
Absorption correction	None
Refinement method	Full-matrix least-squares on F^2
Data / restraints / parameters	2755 / 1 / 301
Goodness-of-fit on F^2	0.871
Final R indices [I>2sigma(I)]	R1 = 0.0377, wR2 = 0.0850
R indices (all data)	R1 = 0.0472, wR2 = 0.0876
Largest diff. peak and hole	0.161 and -0.285 e.A^-3

Reference

[1] Priewisch, B.; Rück-Braun, K. *J. Org. Chem.* **2005**, *70*, 2350–2352.
